# Extremely High Phosphate Sorption Capacity in Cu-Pb-Zn Mine Tailings

**DOI:** 10.1371/journal.pone.0135364

**Published:** 2015-08-21

**Authors:** Longbin Huang, Xiaofang Li, Tuan A. H. Nguyen

**Affiliations:** The University of Queensland, Sustainable Minerals Institute, Centre for Mined Land Rehabilitation, Brisbane, Queensland, 4072, Australia; University of Illinois at Chicago, UNITED STATES

## Abstract

Elevated inorganic phosphate (Pi) concentrations in pore water of amended tailings under direct revegetation may cause toxicity in some native woody species but not native forbs or herb species, all of which are key constituents in target native plant communities for phytostabilizing base metal mine tailings. As a result, Pi sorption capacity has been quantified by a conventional batch procedure in three types of base metal mine tailings sampled from two copper (Cu)-lead (Pb)-zinc (Zn) mines, as the basis for Pi-fertiliser addition. It was found that the Pi-sorption capacity in the tailings and local soil was extremely high, far higher than highly weathered agricultural soils in literature, but similar to those of volcanic ash soils. The Langmuir P-sorption maximum was up to 7.72, 4.12, 4.02 and 3.62 mg P g^-1^ tailings, in the fresh tailings of mixed Cu-Pb-Zn streams (MIMTD7), the weathered tailings of mixed Cu-Pb-Zn streams (MIMTD5), EHM-TD (fresh Cu-stream, high magnetite content) and local soil (weathered shale and schist), respectively. Physicochemical factors highly correlated with the high Pi-sorption in the tailings were fine particle distribution, oxalate and dithionite-citrate-bicarbonate extractable Fe (Fe_O_ and Fe_d_), oxalate-extractable Al and Mn, and the levels of soluble Cd and Zn, and total S and Fe. Large amounts of amorphous Fe oxides and oxyhydroxides may have been formed from the oxidation of pyritic materials and redox cycles of Fe-minerals (such as pyrite (FeS_2_), ankerite (Ca(Fe Mg)(CO_3_)_2_ and siderite (FeCO_3_), as indicated by the extractable Fe_O_ values. The likely formation of sparingly soluble Zn-phosphate in the Pb-Zn tailings containing high levels of Zn (from sphalerite ((Zn,Fe)S, ZnS, (Zn,Cd)S)) may substantially lower soluble Zn levels in the tailings through high rates of Pi-fertiliser addition. As a result, the possibility of P-toxicity in native plant species caused by the addition of soluble phosphate fertilizers would be minimal.

## Introduction

Phytostabilization of base metal (e.g. Cu, Pb, and Zn) mine tailings with native plant communities is one of the sustainable options for the closure of tailings storage facilities [1]. Phosphate (Pi) fertilizer is commonly added in the newly reconstructed root zones for establishing plant cover to stabilize and rehabilitate the metal mine tailings [2]. However, different native plant species have different physiological responses to elevated levels of inorganic phosphate (Pi) in soil solution [3], which may favor some species and/or suppress others in their growth. For example, native herb *Ptilotus* species are highly adaptive to a range of Pi levels from 15 to 100 mg Pi/kg sandy soil [4] and even up to 213 mg soluble P/kg sand in one of our own glasshouse trial in 2013 (unpublished data) with positive biomass growth, while native woody species such as *Acacia chisolmii* expressed P toxicity induced by elevated Pi levels in soil solution in the same treatment of soluble Pi fertilizer application (unpublished data). As both native herbs and woody species are important elements in target plant communities to be rehabilitated, substantial elevation of soluble Pi in soil solution should be avoided when Pi-fertilisers are added into the rehabilitated root zones reconstituted from amended tailings and local soil resources. As a result, it is necessary to understand the Pi-adsorption characteristics in relation to tailings properties, in order to formulate P-fertiliser addition options that are compatible with Pi-acquisition and requirements of native plants and prevent the Pi-toxicity induced species competition among the native plant species in target plant ecosystems to be rehabilitated.

In natural soils, it is well known that phosphorus availability is strongly regulated by P-sorption capacity of soil minerals, which are affected by major soil factors such as Fe-Al oxides content, clay content, pH, and soluble cations (particularly Ca in carbonates and metals) concentration [5–8]. Soil P sorption capacity is positively correlated with amorphous and crystalline Fe/Al oxides in soil, and affected by co-existing anions and cations (especially SO_4_^2-^, Ca^2+^, H^+^) through either competition adsorption or surface precipitation (such as Ca and Mn). High P-sorption capacity is characteristic of soils containing large amounts of Fe/Al oxyhydroxides (acid oxalate- extractable and dithionite citrate bicarbonate (DCB) extractable pools) at acidic pH and Ca/Mg carbonates at neural pH [7, 8].

However, the mineralogy and basic properties of base metal mine (e.g. Cu-Pb-Zn) tailings are very different from those expected in natural soils. Tailings are residue wastes generated from ore processing and finely ground gangue materials, which are rich in many primary minerals present in the tailings such as pyrite, magnetite (Fe_3_O_4_), siderite, calcite (CaCO_3_) and chalcopyrite (CuFeS_2_), etc., which are gradually weathered through chemical and biological processes under local climatic and matrix conditions to form secondary minerals of Ca, Fe, Mn, and other metals in forms of oxides, oxyhydroxides, sulphates and carbonates [9–11]. Large amounts of Fe/Al/Mn oxides and oxyhydroxides, and Ca and Mg are present in the Cu/Zn/Pb mine [11]. In Cu-mine tailings, high levels of calcite and gypsum (CaSO_4_^.^2H_2_O) may also be present [2], which may precipitate soluble P from pore water, thus increasing P sorption [7]. As a result, soluble Pi may be rapidly immobilized through reactions with and adsorption by secondary minerals in the tailings which yield sparsely soluble P-compounds adsorbed or precipitated onto tailings particles [11, 12], thus avoiding P-toxicity risks of elevated soluble Pi in pore water of reconstructed root zones after adding high rates of phosphate fertilisers.

So far little detailed information has been available about the characteristics of Pi sorption in base metal mine tailings, such as Cu-Pb-Zn mine tailings. Limited examples have demonstrated that waste Fe and Al minerals in wastes or mine tailings have very high P-sorption capacity, such as ochre (mainly goethite (FeO(OH)) from coal mine waste water and phlogopite (KMg_3_(Si_3_Al)O_10_(OH)_2_) from apatite processing [13, 14]. The primary objective of the present study is to assess if the base metal mine tailings have very high P-sorption capacity, by characterizing the short-term Pi-sorption behavior of base metal mine tailings, in response to added Pi, as initial information for formulating P-fertilisation strategy and options for tailings revegetation with local native plant species. In the present study, the short-term P sorption behavior of three types of base metal tailings from northwest Queensland has been characterized, in comparison with a local Oxisol soil which is highly mineralized bedrock in the same region. Their P-sorption capacity has been discussed together with P-sorption characteristics of highly P-fixing and natural soils reported in the literature.

## Materials and Methods

The authors had specific permissions from Mount Isa Mines to carry out the study on their private land (mine lease). The head of Environmental Services of Mount Isa Mines should be contacted for future permissions. The bulk tailings and soil samples were collected from Mount Isa (Mt Isa) and Cloncurry region, Northwest Queensland, Australia, where Ernest Henry Mine (EHM) and Mt Isa Mines (MIM) were located. Tailings materials were sampled from the tailings storage facilities (TSF) at EHM (copper (chalcopyrite (CuFeS_2_))-gold mineralization that occurred mainly within the magnetite-biotite-calcite ± pyrite matrix) and MIM (pyrite and chalcopyrite mineralization) in northwest Queensland (unpublished data from Xstrata Copper Australia). MIM tailings were mixtures of Cu and Pb-Zn-Ag streams. Dolomite contents in Cu-tailings were higher than pyrite, but this was the opposite in Zn-Pb tailings (unpublished data from tailings distribution box, provided by Xstrata Copper Australia).

At MIM, the tailings were sampled from the TSFs of different age: TSF No.5 (namely MIM-TD5, about 40 years after decommission) and TSF No.7 (MIM-TD7, still active and receiving mixed streams of fresh tailings). At EHM, the TSF (namely EHM-TD) remained active at sampling, receiving tailings stream from Cu-ore processing. From visual observation at sampling, the oxidation zone in TD5 had progressed to >100 cm in depth. In contrast, TD7 contained freshly deposited tailings from mixed streams of Zn, Pb and Cu ore processing, without distinct oxidised zone in the TD7 tailings profile, due to continuous inputs of fresh tailings slurry and wastewater. For comparison, a natural soil (top soil) was collected from a hillside (HS) adjacent to TD5. The bulk tailings and soil samples were dried at 40°C in an air-drafted oven until constant weight and sieved through 1 mm mesh by a stainless steel sieve. The sieved samples were stored in sealed plastic bags at room temperature prior to laboratory tests and analysis.

### Physicochemical analysis

The air-dry and sieved samples were used for the analysis of particle size distribution in the range of 0.1–2000 μm by using laser diffraction (Mastersizer 2000, Malvern Instruments Ltd, UK). The pH of the tailings and soil samples were measured in water extracts at a solid:solution ratio of 1:5 (w/v), after being mixed on an end-over-end shaker for 1 hour at room temperature by using a pH electrode (TPS 900-P) [15].

Total concentrations of cation and metal elements were determined by means of an Inductively Coupled Plasma Optical Emission Spectroscopy (ICP-OES) (Varian Vista Liberty, Australia) after aqua-regia acid digestion [16]. Standard reference soil samples (NIST2709) were also included to assure correct dissolution and analysis of the studied samples. Briefly, about 1 g aliquots of air-dried tailings were digested in 20 ml concentrated nitric-hydrochloric acid mix (3:2, v/v) in pressurized Teflon vessels which were heated by microwave heating at 180°C for 20 min. After cooling, the digest was made up to 50 ml final volume using DI water.

The air-dried tailings/soil samples were extracted for acid-oxalate and DCB-extractable Fe and Al [15]. For acid-oxalate-extractable Fe/Al, 1.0 g tailings/soil was shaken with 100 ml acid oxalate in the dark for 4h at 25°C. For DCB-extractable Fe/Al, 1.0 g tailings/soil was shaken with 50 ml 22% sodium citrate and 1.0 g sodium dithionite for 16h at 25°C. Then 50 ml DI water and 5 drops of Superfloc solution were added and shook vigorously for 5s. All the clear supernatants were filtered through 0.45 μm filter before ICP-OES analysis. The acid-oxalate-extractable Fe/Al is supposed to include amorphous Fe (such as ferrihydrite (Fe_5_HO_8_^.^4H_2_O)) and Al, organically complexed Fe and Al and Fe minerals containing Fe^2+^ such as magnetite [17, 18]. DCB-extractable Fe/Al is supposed to include oxides and hydroxides of Fe (such as hematite, goethite in soil, and if present, lepidocrocite (gamma-FeOOH) and Maghemite (gamma-Fe_2_O_3_), magnetite, and ferrihydrite) and Al [18].

### Phosphate sorption isotherms

Phosphate sorption in the tailings and soil samples were evaluated from short-term sorption tests using protocols modified from Wisawapipat et al. [19]. Briefly, 1g air-dried and sieved (< 1mm) tailings or soil was equilibrated with 50 mL phosphate solution in disposable centrifuge tubes (50 ml), containing initial Pi concentrations (mg P/L): 0, 23.8, 48.0, 96.0, 191.9, 400, 800, 1200, 1600, and 2000. The phosphate solutions were prepared by dissolving di-ammonium hydrogen phosphate (AR grade) in Millipore water. Due to the high levels of Ca and Mg already present in the tailings, no background electrolyte was used in the P-solutions. Each P concentration was replicated 3 times. This range of P concentrations was selected based on preliminary experiments (data not shown). The mixture samples were shaken for 24 h at room temperature (around 20°C) on an end-over-end shaker. At the end of the shaking period, the sample mixture solutions were centrifuged at 4000 g (Eppendorf Centrifuge 5810) for 10 min and the supernatants were further filtered through 0.45 μm filter. Residual soluble P (mostly inorganic Pi) concentration was then determined by the spectrophotometric molybdenum blue method [15].

### Particle size distribution and XRD analysis

Particle size distribution in the samples was quantified using laser diffraction method, following the standard protocol for the machine (Mastersizer 2000, Malvern Instruments Ltd, UK). Dry and sieved samples (<1 mm sieved samples) were weighed for XRD analysis using a Bruker D8 Advance X-Ray Diffractometer equipped with a scintillation counter, graphite monochromator and copper target (Bruker Corporation). The XRD operational conditions were as follows: 40 kV generator tension, 30 mA generator current, from 2 to 70 degrees 2-Theta, 0.050 degree step size, 20mm variable slits and 0.5 second per step. Prominent minerals (reporting level of differences in mineral phase composition was 1%) were identified by EVA software with its embedded database.

### Data analysis

Phosphorus sorption in each sample was calculated as the difference between initial P and residual P concentrations in solution. The Langmuir and Freundlich models were used to fit the phosphorous adsorption isotherm of the base metal mine tailings and the soil samples [5, 8, 20]. The Langmuir model describes the surface as homogeneous assuming that all the adsorption sites have equal adsorbate affinity and that adsorption at one site does not affect adsorption at an adjacent site. The equation of the Langmuir model is expressed: *x* = *X*_*m*_ *bc*/(1+*bc*); where *x* is the amount of P adsorbed (mg P g^-1^ tailings or soil), *X*_*m*_ is Langmuir maximum sorption capacity (mg P g^-1^), *b* is the constant related to the affinity (L mg^-1^ P), and *c* is P concentration in equilibrium solution (mg P L^-1^),.

On the other hand, the Freundlich model describes the adsorption on heterogeneous surfaces and does not assume monolayer capacity. The Freundlich equation is shown: *x = kc* ^*B*^, where *k* (mg^1-B^ L^B^ g^-1^) and *B* are empirical coefficients indicating adsorption capacity and intensity, respectively.

Statistical tests using LSD (*P ≤* 0.05) was conducted on the data of total elemental concentrations and extractable Al, Fe and Mn in the samples to detect if the means were significantly different from each other. Simple correlation analysis was carried out between Pi-sorption capacity and physical and chemical properties of the tailings.

## Results

### Total elemental concentration and DCB- and oxalate-extractable Al, Fe and Mn

Total elemental (e.g. Al, Ca, Fe, Mn) and oxalate- and dithionite- extractable Al, Fe and Mn concentrations in the samples were determined to evaluate the relationship of mineral elements and their amorphous/poorly crystalline forms with the Pi-adsorption capacity. Generally, the tailings contained high concentrations of total Al, Ca, and Fe, and base metals (e.g. Cu, Pb, Zn), which are candidates of P-sorption, compared with the HS soil (Table 1). In particular, total Fe concentrations in MIM-TD7 and EHM-TD were nearly twice as much as those in MIM-TD5 tailings and HS soil. Total Al concentrations in the samples were significantly different from each other, with the order of HS soil > EHM-TD > MIM-TD7 > MIM-TD5. MIM-TD7 contained the highest total S concentration, followed by MIM-TD5, EHM-TD and HS soil (Table 1), which coincided with its lowest pH (Table 2). Total Ca concentrations followed the order of MIM-TD5 >> MIM-TD7 ≈ EHM-TD >> HS. Total Mn concentrations in EHM-TD and MIM-TD5 were similarly higher than that in MIM-TD7, with the lowest in HS soil (Table 1). EHM-TD, MIM-TD5 and MIM-TD7 tailings exhibited average pH of 8.3, 8.2 and 6.7 respectively, while the HS soil 8.3 (Table 2).

**Table 1:** Total element concentrations in the tailings and hillside soil samples. The values are means of 3 replicates each, with standard deviations in the parentheses.

Sample	Al (mg g^-1^)	As (mg kg^-1^)	Ca (mg g^-1^)	Cd (mg kg^-1^)	Fe (mg g^-1^)	P (mg kg^-1^)	S (mg g^-1^)	Mn (mg g^-1^)	Cu (mg kg^-1^)	Pb (mg kg^-1^)	Zn (mg kg^-1^)
**EHM-TD**	52.4 ^B^	95.1	10.3 ^C^	0.355^B^	128 ^A^	973 ^A^	10.8 ^C^	1.64 ^A^	383 ^B^	19.3 ^C^	27.1^C^
	(3.95)	(4.84)	(0.26)	(0.228)	(15.1)	(71)	(1.00)	(0.17)	(123)	(9.44)	(2.16)
**MIM-TD5**	5.01 ^D^	242	48.7 ^A^	8.74 ^C^	60.2 ^C^	308 ^B^	33.2 ^B^	1.61^A^	1309 ^A^	1825 ^B^	2896 ^B^
	(0.50)	(8.78)	(0.88)	(0.44)	(2.06)	(21)	(0.22)	(0.06)	(32.3)	(23.5)	(114)
**MIM-TD7**	18.5 ^C^	1018	17.8 ^B^	27.5^D^	109 ^B^	370 ^B^	64.8 ^A^	0.98 ^B^	1711^A^	3902 ^A^	8504 ^A^
	(1.03)	(6.56)	(0.41)	(1.29)	(4.51)	(7.0)	(2.40)	(0.02)	(78.6)	(28.7)	(215)
**HS**	66.0^A^	8.76	4.19 ^D^	0.165^A^	54.4 ^D^	104 ^C^	0.310 ^D^	0.39 ^C^	64.9 ^C^	37.6^C^	64.2 ^C^
	(4.35)	(0.80)	(1.84)	(0.070)	(2.46)	(12)	(0.11)	(0.01)	(3.66)	(19.3)	(29.1)

For the same element, different letters indicated the significant difference at P ≤ 0.05.

**Table 2:** Chemical forms of Al, Fe and Mn in tailings and Hillside soil samples. In the table, Al_O_, Fe_O_ and Mn_O_ are oxalate-extractable Al, Fe and Mn (non-crystalline form). Al_d_, Fe_d_ and Mn_d_ are dithionite-citrate-bicarbonate extractable fractions (crystalline form). The values were means of 3 replicates per sample with standard deviation in the parentheses.

Sample name	pH_w_(1:5)	Al_O_	Al_d_	Fe_O_	Fe_d_	Mn_O_	Mn_d_
		*(g/kg dry weight)*					
**EHM-TD**	8.3	0.56 ^B^ (0.02)	0.27 ^C^ (0.04)	18.51 ^B^ (4.65)	11.28 ^B^ (1.73)	0.27 ^B^ (0.03)	1.28 ^A^ (0.12)
**MIM-TD5**	8.2	0.16 ^D^ (0.02)	0.04 ^D^ (0.01)	15.60^B^ (2.50)	13.88 ^AB^ (0.93)	0.89 ^A^ (0.13)	0.44 ^B^ (0.01)
**MIM-TD7**	6.7	0.67 ^A^ (0.04)	0.42 ^B^ (0.02)	22.95 ^A^ (1.84)	16.58 ^A^ (0.36)	0.93 ^A^ (0.06)	0.39 ^B^ (0.02)
**HS**	8.3	0.34 ^C^ (0.01)	0.61 ^A^ (0.20)	0.40 ^C^ (0.04)	13.58 ^B^ (3.15)	0.18 ^B^ (0.01)	0.23 ^C^ (0.02)

For the same element, different letters indicated the significant difference at P ≤ 0.05.

The tailings and the HS soil contained different amounts of extractable Al, Fe and Mn (Table 2). MIM-TD7 contained the highest concentration of oxalate-extractable Al (Al_O_), followed by EHM-TD > HS > MIM-TD5. However, the HS soil contained the highest concentration of DCB-extractable Al (Al_d_), followed by MIM-TD7 > EHM-TD, with the lowest in MIM-TD5. The level of oxalate-extractable Fe (Fe_O_) was highest in MIM-TD7, followed by EHM-TD and MIM-TD5, with the lowest in the HS soil (Table 2). The DCB-Fe (Fe_d_) concentrations were similar between MIM-TD5 and MIM-TD7, which were higher than that of EHM-TD. The Fe_d_ concentration in the HS was similar to EHM-TD and MIM-TD5. MIM-TD5 and MIM-TD7 contained similar amounts of oxalate-extractable Mn (Mn_O_), which were higher than those in EHM-TD and HS soil. In comparison, EHM-TD contained the highest Mn_d_ concentration and the HS lowest, while MIM-TD5 and MIM-TD7 had similar amounts of Mn_d_ (Table 2).

### Phosphate sorption capacity

**Fig 1:**
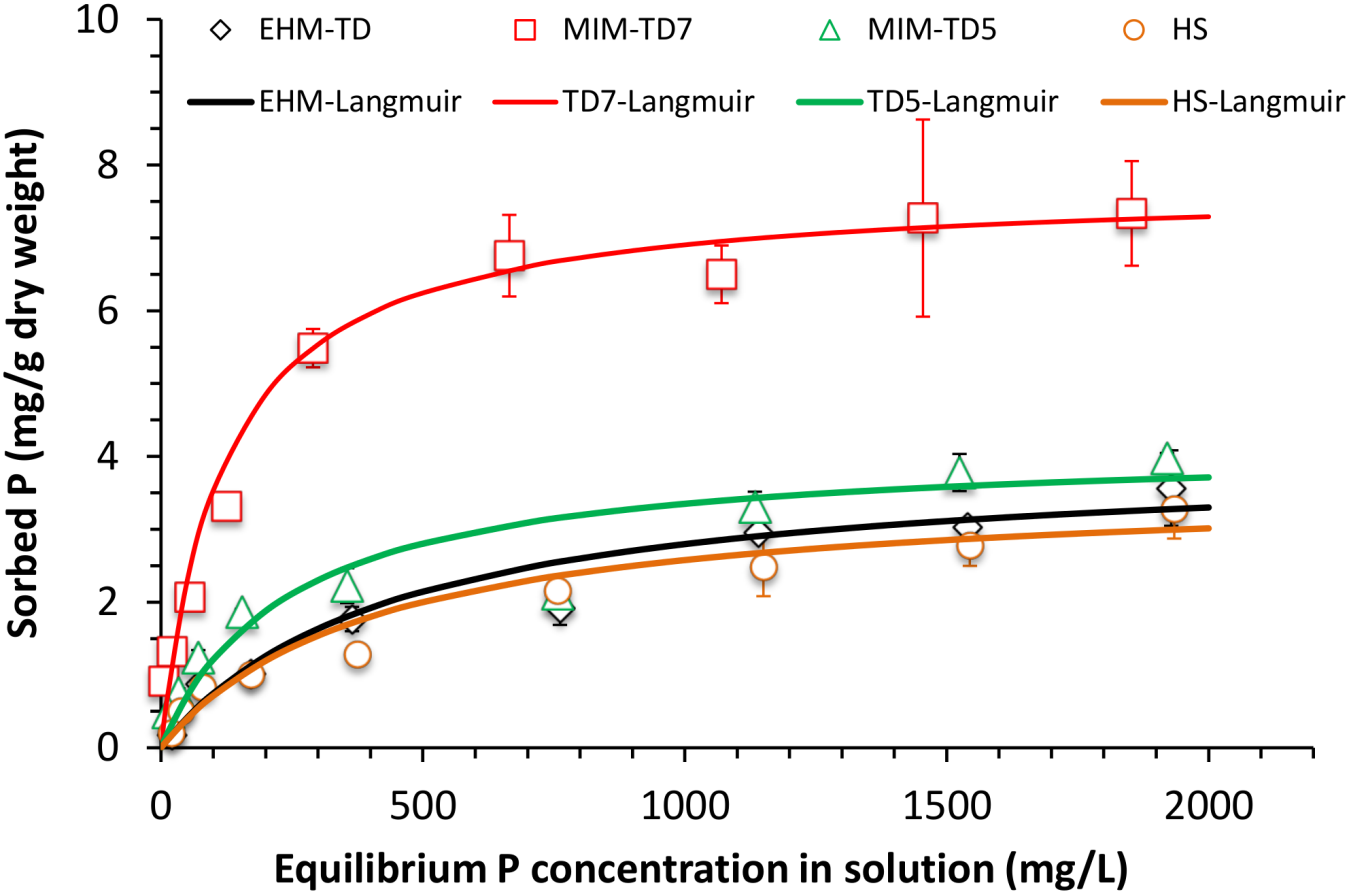
Phosphate sorption isotherms in the tailings and HS soil, which were fitted with Langmuir equation (see Table 3). The values were averages of 3 replicates at each P-concentration and the bars indicate corresponding standard deviation.

For understanding the relationship between the Pi-adsorption behavior and physicochemical properties of the tailings and HS samples, the maximal Pi-adsorption capacity was estimated by using Langmuir and Freundlich equations. Both equations closely described the relationships between equilibrium P concentrations in solution and P-sorbed by the tailings and soil samples, with *R* ^*2*^ 0.93–0.99 for Langmuir and 0.94–0.99 for Freundlich (Table 3). In general, MIM tailings materials exhibited stronger P-sorption capacity than EHM and local soil (HS) (Fig 1, Table 3). On the basis of the maximum P-sorption estimated from Langmuir equation, MIM-TD7 had the highest P-sorption capacity (7.72 mg P/g air-dry weight), which was much higher than those of MIM-TD5. The P-sorption capacity of the HS soil was comparable to those of EHM-TD tailings (Table 3). The estimated P-sorption capacity in these samples were far higher than those crop soils reported in literature (Table 4), but was generally similar to those of volcanic ash soils (Table 4).

**Table 3:** Nonlinear regression analysis of the relationship between equilibrium P concentration in solution and P adsorbed by the tailings or soil, based on Langmuir and Freundlich equations. Langmuir—X_m_, the P-sorption maximum; *b* related to bonding energy; Freundlich—*k* and *B* are empirical coefficients: *k* is the sorptivity (mL g^-1^), an index of sorption capacity and *B* is related to bonding energy.

Sample name	Langmuir			Freundlich		
	X_*m*_ (mg g^-1^)	*b* (mL mg^-1^)	R^2^	*K* (mg^1-B^ L^B^ g^-1^)	B	R^2^
EHM-TD	4.02	2.28	0.94	0.08	0.49	0.98
MIM-TD5	4.16	4.12	0.93	0.23	0.38	0.95
MIM-TD7	7.72	8.45	0.99	0.73	0.32	0.94
HS	3.62	2.46	0.95	0.08	0.49	0.99

Note: EHM = Ernest Henry Mine, TD5 and TD7 = MIM-Tailings Dam 5 and 7; HS = Hillside topsoil; LTD5 = leached TD5 tailings before adsorption test.

**Table 4:** Langmuir P maximum values and contents of extractable Al and Fe reported in the literature.

Country/Region	Soil type	Maximum Langmuir P sorbed (mg g^-1^)	Extractable and total Fe, Al (g kg^-1^ dry weight) (±SD)						References
			Fe_o_	Fe_d_	Fe_t_	Al_o_	Al_d_	Al_t_	
Alaska, US	Volcanic ash soils	10.12	8.8–10.9	9.1–15.3	ND	9.2–14.3	5.4–10.7	ND	[22]
	Loess soils	3.93	5.6–8.7	8.2–14.6	ND	1.4–2.8	2.1–11.0	ND	[22]
Indonesia	Andosols (Volcanic ash)	4.51	ND	ND	ND	ND	ND	ND	[23]
Upland, Thailand	Shale/limestone soils	0.17–0.83	2.3±0.66	78±28	143±78	5.5±3.9	5.1±8.0	316±97	[19]
	Basaltic soils	0.46–1.25	14±7.4	94±25	186±66	7.3±4.4	20±8.5	280±20	[19]
	Granitic soils	0.05–0.40	1.3±0.77	5.0±7.0	11±14	0.83±0.62	1.8±2.1	60±70	[19]
	Old alluvium & sediments	0.05–0.50	1.1±0.65	6.3±6.2	12±12	2.5±1.5	3.1±1.9	39±51	[19]
South western Australia	Soils with Hematite	0.62	1.038	21.71	ND	2.18	3.94	ND	[8]
	Soils without Hematite	0.07	0.425	2.01	ND	0.643	0.883	ND	[8]
	Soils with Goethite	0.27	0.724	10.01	ND	0.724	2.742	ND	[8]
	Soils without Goethite	0.14	0.290	0.855	ND	0.290	0.461	ND	[8]

Notes: Fe_o_ or Al_o_ are Fe and Al extracted by acid oxalate; Fe_d_ or Al_d_ are Fe and Al extracted by dithionite-citrate-bicarbonate solution; Fe_t_ or Al_t_ are total Fe and Al. SD = Standard devidation; ND = not determined.

### Particle size distribution

**Fig 2:**
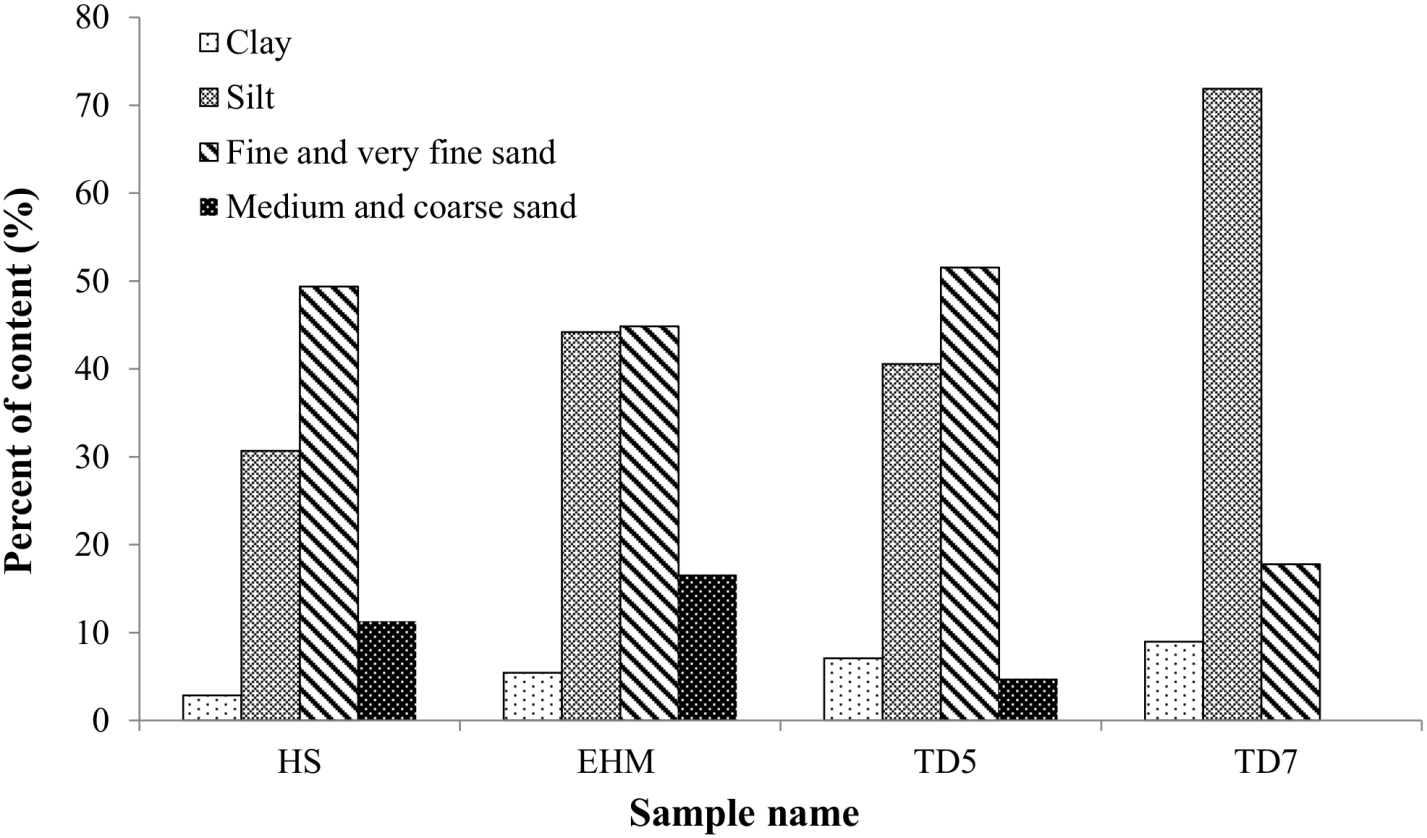
The particle size distribution of the tailings samples and HS soil sample.

The surface area is one of the critical physical properties in determining Pi-adsorption by mineral particles in the tailings and HS sample. The particle size distribution differed substantially among the samples (Fig 2). EHM-TD, HS and MIM-TD5 were similar in texture, based on the proportions of particle size distribution. However, MIM-TD7 contained the highest distribution of fine particles (0.002–0.05 mm, equivalent to silt-fine silt fractions). The HS sample had the lowest distribution of silt fractions.

### Mineralogy

The weathering and transformation of unstable primary minerals give rise to secondary minerals (e.g. Al-, Ca-, Fe- Mn-, and/or Zn-minerals) with high Pi-adsorption. As a result, the XRD analysis was conducted to reveal different patterns of minerals present in the tailings and natural soil (Table 5). The minerals present in the tailings and HS samples can be grouped into three groups: Ca/Mg-, Fe-, and Al-minerals, which are candidates of P-sorption. Among the Ca-minerals, calcite was present in EHM-TD, but dolomite and gypsum in MIM-TD7, and both calcite, gypsum and dolomite in MIM-TD5. These calcium minerals were not detected in the HS soil sample. Among the Fe-minerals, EHM-TD contained both magnetite and pyrite (Table 5), but quantitative XRD analysis showed about 29% magnetite present in EHM-TD tailings [21]. Minerals present in MIM-TD5 include pyrite, ankerite, and siderite and in MIM-TD7 mainly pyrite, ankerite and sphalerite. However, the HS soil mainly contained crystalline Fe minerals, goethite and hematite (α–Fe_2_O_3_).

**Table 5:** The presence of major primary and secondary minerals identified in the tailings and soil samples by XRD-analysis.

Mineral	Dominant Composition	EHM-TD	MIM-TD5	MIM-TD7	HS
Quartz	SiO_2_	√	√	√	√
Calcite	Ca(CO_3_)_2_	√	√		
Dolomite	CaMg(CO_3_)_2_		√	√	
Gypsum	CaSO_4_(H_2_O)_2_		√	√	
Ankerite	Ca(Fe^2+^,Mg,Mn^2+^)(CO_3_)_2_		√		
Pyrite	FeS_2_	√	√	√	
Siderite	Fe^2+^CO_3_		√	√	
Sphalerite	(Zn,Fe)S			√	
Goethite	α–Fe^3+^O(OH)				√
Hematite	α–Fe_2_O_3_				√
Magnetite	Fe_3_O_4_	√			
Linarite	PbCu(SO_4_)(OH)_2_			√	
Kaolinite	Al_2_Si_2_O_5_(OH)_4_	√	√	√	
Muscovite	KAl_2_(Si_3_Al)O10(OH;F)_2_			√	√
Microcline	KAlSi_3_O_8_	√			
Orthoclase	KAlSi_3_O_8_	√			

A range of Al-Si minerals were found in the tailings samples (Table 5): microcline (KAlSi_3_O_8_), orthoclase (KAlSi_3_O_8_) and kaolinite (Al_2_Si_2_O_5_(OH)_4_) are present in EHM-TD, but kaolinite and muscovite (KAl_2_(Si_3_Al)O_10_(OH;F)_2_) only in MIM-TD7. Linarite (PbCu(SO_4_)(OH)_2_) was also present in MIM-TD7 sample. The HS soil contained mainly muscovite.

### Correlations between Pi-sorption capacity and physical and chemical properties

Many physical and chemical properties of the tailings and soil contributed to the total Pi-sorption capacity, with different proportions (Table 6). The Pi-sorption capacity was highly correlated with the proportions of fine particle fractions including clay and silt (fine-coarse), with coefficients 0.67–0.99. Both Fe_O_ and Fe_d_ significantly contributed to the Pi-sorption capacity, with coefficients of 0.67 and 0.84 respectively. The Al_O_ and Mn_O_ also correlated with the Pi-sorption with coefficients of 0.66 and 0.68, respectively. Interestingly the soluble Cd and Zn in the tailings had a close and positive correlationship with Pi-sorption with a coefficient value >0.90, which were from the weathering of Sphalerite minerals (e.g. (Zn,Cd)S) in the tailings. Among the total elemental concentrations, the concentration of total S was closely related to the Pi-sorption with a coefficient of 0.93, perhaps indicating the degree of oxidation of pyrite and thus the formation of Fe oxides/oxyhydroxides.

**Table 6:** Correlation coefficient (*C)* values between phosphate sorption capacity and selected physical and chemical properties of the tailings and local soil.

C											
**Particle size distribution**	Clay	0.83	**Extractable Al, Fe, Mn**	Al_d_	0.10	**Soluble metals & SO**_**4**_ **-S**	Cd	0.97	**Total element**	Fe	0.41
	Fine silt	0.99		Fe_d_	0.84		Cu	0.05		S	0.93
	Medium silt	0.98		Mn_d_	-0.23		Ni	0.19		Al	-0.51
	Coarse silt	0.67		Al_O_	0.66		Pb	0.24		Ca	0.03
	Fine sand	-0.83		Fe_O_	0.67		Zn	0.99		K	-0.31
	Medium sand	-0.92		Mn_O_	0.68		S	0.01		Mg	-0.05
	Coarse sand	-0.31								Na	-0.34
										P	-0.07
										Mn	-0.07

## Discussion

The present results have confirmed that Pi-sorption capacity in the base metal mine tailings from mixed streams of ore processing of Cu, Pb and Zn mines were extremely high, ranging from 3.62 to 7.72 mg Pi g^-1^ air-dry weight, rendering the low risk of elevated soluble Pi in pore water of these materials after soluble Pi-fertilizers are added in reconstructed root zones for revegetation with native plant communities. Previous studies have shown that mine wastes containing high contents of Fe and Al minerals can retain very high loads of Pi, such as orchre (particle size 0.25–10 mm) from coal mine wastewater with a maximal Pi sorption of 22.78 mg Pi g^-1^ dry weight and phlogopite from apatite ore processing (pH 3–5, size < 0.2 mm) with a sorption capacity of 11.42–14.42 mg Pi g^-1^ dry weight [13, 14]. This kind of Pi-sorption capacity is far greater than those in cropping and forestry soils, even those shale and basaltic soils with high Fe contents (Table 4), but in a similar category as volcanic ash and loess soils [22, 23]. A local soil collected from an adjacent native vegetation site also exhibited a very high Pi-sorption capacity, in comparison with natural soils under crops or forests (Table 4), with *X*_m_ similar to that in EHM tailing that is of slightly alkaline pH and high magnetite content. Since the tailings are simply ground rock ores and gangue materials, they were in the initial phase of rapid oxidation and weathering of Al, Ca and/or Fe-containing minerals. The local soil was formed from weathered rock fragments and particles (such as shale, schist and limestone), with much longer history than the tailings [24, 25]. From the correlation analysis, key physical and chemical factors in the tailings and local soil may include fine particle distribution (clay and silt fractions), extractable Fe (Fe_O_ and Fe_d_), Al_O_ and Mn_O_, and the levels of soluble Cd and Zn, and total S and Fe, which were from the weathering of sphalerite, pyrite, and chalcopyrite minerals.

The high correlation between Pi-sorption capacity and the proportions of particles of clay and silt sizes suggests the important contribution of high specific surface area toward the Pi-sorption process, since the surface area per unit volume (or mass) increases as particle size decreases. The order of clay and silt distribution among the tailings and local soil followed the order of their Pi-sorption capacity, with the highest proportion of silt particles and Pi-sorption in the MIM-TD7 tailings. The much finer particles in the MIM-TD7 tailings resulted from much improved grinding efficiency in modern ore processing technology, in comparison with the MIM-TD5 which was subject to about 40 years of weathering. However, the presence of high specific surface area alone is not adequate to explain the high Pi-sorption capacity in the tailings and local soil. Chemical affinity for Pi must be present on the particle surfaces to form Pi-mineral complex and/or surface precipitates (at high concentrations) [20, 26]. At a similar surface area, the Pi-adsorption would increase with increasing density of high Pi-adsorbing secondary minerals which are (co-)precipitated onto the surfaces of tailings particles.

The tailings used in the present study contained abundant Ca-minerals (such as calcite and dolomite) and Fe- minerals (such as pyrite, ankerite and siderite), with variations among the tailings, which have been subject to complex geochemical processes, including oxidation/reduction and dissolution/precipitation under subtropical and semi-arid climatic conditions. The Fe-minerals were dominated by pyrite (6–35%) in MIM-TD7, but magnetite (20–30%) in EHM tailings (with some pyrite and chalcopyrite) [10, 21]. Large amounts of amorphous secondary Fe minerals may have been formed from the primary Fe-containing minerals, which were deposited as mineral granules and/or coated the particles of primary Fe-minerals in tailings, thus providing sorption sites for Pi. The Fe-containing minerals (including pyrite, arsenopyrite (AsFeS), and chalcolpyrite) deposited at surface layer can undergo rapid oxidation upon exposure to oxygen and water, leading to the formation of fine grained Fe(III) hydroxide rich, ochre to red-brown colored profile in the tailings [9, 10, 27]. In fact, red-brown, rusty profile was observed by the authors in the first 50–100 cm tailings profile when collecting the MIM-TD5 tailings, but the MIM-TD7 tailings appear dark grey in color, with rapid formation of sulfate salt crust upon dewatering and exposure to the air.

The primary Fe-minerals alone cannot be adequately accounted for the extremely high Pi-sorption capacity, without the formation of large amounts of amorphous Fe oxides and oxyhydroxides in the tailings. By comparison, ferrihydrite or hydrous ferric oxides have much higher surface area and affinity for Pi than magnetite and lepidocrocite, adsorbing 10 times more Pi than the latter [28]. The oxidation of pyritic minerals in the MIM-TD5 and TD7 were verified by the presence of large amounts of sulfate in pore water of tailings and seepage water from both the TD5 and TD7 tailings [10, 29]. In fact, an earlier study with the aged TD5 tailings, revealed the presence of large amounts of hydrous ferric oxide conglomerate with biotite [9, 10]. Bioreduction or biomineralization of Fe-minerals can be facilitated by dissimilatory metal reducing bacteria which can transform Fe(III) into Fe(II), leading to the formation of many secondary Fe oxides and oxyhydroxides which provide high affinity sites for Pi sorption [30, 31]. In addition, in a recent incubation trial with EHM tailings (the same as used in the present study) by the authors, large amounts of soluble Fe (II) was recovered in the pore water of the tailings under well-watered conditions, with soluble Fe concentrations up to 50 mg L^-1^ (unpublished data). The presence of abundant amorphous Fe oxides and oxyhydroxides in the tailings was in line with high levels of Fe_O_, which were substantially higher than most of agricultural soils, but compatible with those in volcanic ash and loess soils of extremely high Pi-sorption capacity [22, 23]. As a result, it is reasonable to conclude that the combination of abundant amorphous Fe oxides and oxyhydroxides and fine texture (high surface area relative to natural soil) would have at least, substantially contributed to the extremely high Pi-sorption capacity in general, in the base metal mine tailings.

In weathered soils, the levels of oxalate extractable Fe are usually lower than the DCB-extractable Fe, therefore, the Fe_O_/Fe_d_ ratio is expected low (<1) due to the high degree of crystallinity in Fe oxides (see Table 4 for references). However, in the present tailings, the amount of Fe_O_ was higher than that of Fe_d_. The possibility of extraction error was ruled out by repeated analysis of the same samples in the present study and later analysis in separate studies. The acid-oxalate-extractable Fe/Al is supposed to include amorphous Fe (such as ferrihydrite) and Al, organically complexed Fe, and Al and Fe minerals containing Fe^2+^ such as magnetite [17, 18]. DCB-extractable Fe/Al is supposed to include oxides and hydroxides of Fe (such as hematite, goethite in soil, and if present, lepidocrocite, maghemite, magnetite, and ferrihydrite) and Al [18]. It is noteworthy that acid oxalate extractant can solubilize Fe^2+^ in magnetite, pyrite, siderite and ankerite, which may cause an over-estimation of oxalate-extractable Al_O_ and Fe_O_ [32]. Similar levels of Fe_O_ and Fe_d_ were observed in volcanic ash soils [22]. The pattern of Fe_O_ > Fe_d_ has also been observed in Andisols which are rich in ferrihydrite [33] and buried paleosols [32]. In fact, the local soil had a pattern of Fe_O_ < Fe_d_, similar to other patterns reported in weathered soils (see Table 4 for reference). As a result, the high Fe_O_/Fe_d_ ratios in these studied tailings were most likely attributed to the newly formed Fe-oxyhydroxides from the weathering of tailings minerals that were yet to be converted into crystalline Fe, as the tailings were much less weathered than natural soil.

Large amounts of amorphous Fe oxides and oxyhydroxides may have been formed from oxidation of Fe-containing primary minerals (as described in earlier sections) in the tailings which were undergoing weathering in periodical wet-dry cycles under the subtropical and semi-arid climatic conditions in Northwest Queensland. This was also observed in other studies with the same tailings [9, 10]. These amorphous Fe minerals which exhibit high Pi-adsorption affinity, have yet been converted into highly crystalline forms which requires lengthy period of weathering time. In natural soil, amorphous Fe is gradually converted into crystalline Fe after long-term pedogenesis [32, 34]. The ratios of Fe_O_ to Fe_d_ (as an index of the degree of crystallinity [35, 36] in the tailings investigated here are in a similar category to those in volcanic ash soils, but much higher than those in agricultural soils with high weathering (e.g. granitic and lateritic soils) (see Table 5). It is also worth noting that, under mine tailing storage conditions, surfaces of ferrihydrite may be deposited with phosphate, silicate, and arsenate. These surface impurities inhibit the transformation of ferrihydrite to more crystalline phases such as hematite and goethite. Further investigation is warranted to characterize the dynamic processes of weathering and transformation of Fe-minerals in the tailings by means of X-ray absorption spectroscopy and mineral liberation analysis [37, 38].

The presence of Ca/Mg-minerals (e.g. gypsum, calcite, dolomite, Ankerite) would have also contributed to the P-sorption in the tailings, through Ca/Mg-Phosphate co-precipitation at neutral-slightly alkaline pH. It is well known that phosphate anion can be rapidly sorbed on Ca-carbonate surfaces at low Pi concentration and form precipitates of Ca-phosphate at high Pi concentrations at neutral pH [6, 39]. Total concentrations of Ca and Mg were much higher in the tailings, than the local soil (HS), particularly the Cu-tailings (EHM-TD and MIM-TD5) with 2–5% Ca and 0.5–2% Mg (Table 1). The water-solubility (mg/100 ml water at 20°C) of Ca- and Mg-phosphates is very low, about 2 and 0.26, respectively. There were abundant amounts of Ca and Mg salts in the surface of oxidized tailings, which was visually observed as salt efflorescence in the field by the authors. Phosphate reactions with Ca and Mg would contribute to the total P-sorption of the tailings, particularly in neutral and alkaline pH conditions. The readiness of this fraction P for the uptake of native plant species remain to be investigated in future experiments.

High values of the correlation coefficient R^2^ (<0.93, Table 3) suggest that both adsorption models gave generally good fit to the Pi-sorption isotherms. More information on the surface properties of tailings can be gained by analyzing parameters K and 1/B of the Freundlich model. In general, K, being specific for each adsorbate, is higher for a higher adsorption capacity of the adsorbent. The latter parameter, 1/B, reflects the impact of surface heterogeneity on the adsorption, by which a value closer to 1 could assume a less important role of surface heterogeneity. As 1/B approaches 10, surface heterogeneity plays an important role on the adsorption capacity and intensity. The fresh tailing TD7 was predicted having the largest 1/B, following by TD5. The heterogeneity on the surface of TD7 mineral particles could come from its high content of amorphous phases (Table 2), resulting in much higher Pi adsorption capacity than other samples. This is also in good agreement with the order of the affinity coefficient b obtained from the Langmuir model. Among the studied samples, TD7 had the highest affinity for Pi (b = 8.45). The affinity of TD5 to Pi is about 50% less than TD7, while EHM sample is less attractive to Pi.

The formation of phosphate precipitates with Cd and Zn may have also contributed towards the P-sorption capacity in the tailings, as indicated by the highly positive correlationship. The sphalerite in the Pb-Zn tailings can be oxidized to release large amounts of Cd and Zn into pore water, though the sphalerite was dominated by ZnS and (Zn,Fe)S forms rather than (Zn,Cd)S [9, 10]. The formation of Zn-phosphate is a pathway of lowering soluble Zn levels in soil solutions as it is insoluble in water at neutral pH [40]. As a result, the application of phosphate fertilizers in base metal mine tailings may have additional benefits of lowering toxic metal levels in pore water.

## Conclusion

From the results, it is concluded that base metal mine tailings and highly mineralized soils from weathered rocks have extremely high Pi-sorption capacity, due to combined effects of fine texture (mainly silt fraction) and high levels of newly formed amorphous Fe oxides and oxyhydroxides from weathering of Fe-containing primary minerals and transformation in response to seasonal wet-prolonged dry cycles under the subtropical and semi-arid climatic conditions. The high Pi adsorption of the tailings may be controlled by the physical factor of high surface area of fine particles which are precipitated/coated with different densities of high Pi-adsorbing secondary minerals (mainly amorphous Fe-oxyhydroxides and other minerals (e.g. gypsum)) formed from the weathering of unstable primary minerals. As a result, the added soluble P-fertilizers can be rapidly sorbed and immobilized by tailings solid phase, without high risks of elevated Pi concentrations in pore water within reconstructed root zones from amended tailings and local topsoils.

## Supporting Information

S1 Dataset The dataset contains all the data used to prepare Figs 1 and 2, and Tables 1 and 3. (XLSX)
